# Heterochrony in the evolution of Trinidadian guppy offspring size: maturation along a uniform ontogenetic trajectory

**DOI:** 10.1098/rspb.2017.1319

**Published:** 2017-10-11

**Authors:** T. R. Dial, D. N. Reznick, E. L. Brainerd

**Affiliations:** 1 Department of Ecology and Evolutionary Biology, Brown University, Providence, RI, USA; 2 Department of Biology, University of California, Riverside, CA, USA

**Keywords:** allometry, development, heterochrony, maturity, offspring, ossification

## Abstract

The size and maturity of Trinidadian guppy (*Poecilia reticulata*) offspring vary among populations adapted to environments of differential predation. Guppy offspring born to low-predation, high-competition environments are larger and more mature than their high-predation ancestors. Here we ask: what specific changes in developmental or birth timing occur to produce the larger, more mature neonates? We collected specimens across the perinatal window of development from five populations and quantified musculoskeletal maturation. We found that all populations undergo similar ontogenetic trajectories in skeletal and muscle acquisition; the only difference among populations is when neonates emerge along the trajectory. The smallest neonates are born with 20% of their skeleton ossified, whereas the largest neonates are born with over 70% of their skeleton ossified. The area of the major jaw-closing muscle is relatively larger in larger offspring, scaling with length as L^2.5^. The size range over which offspring are birthed among populations sits along the steepest part of the size–maturity relationship, which provides a large marginal increase in fitness for the high-competition female. Because of the functional effects of producing more mature offspring at birth, offspring size may be the first and most critical life-history trait selected upon in highly competitive environments.

## 1. Introduction

The evolution of life histories can influence organismal form and function through mechanistic changes in the timing or rate of size or shape acquisition [1]. In passerine birds, for example, strong nest predation at lower latitudes selects for early fledging by favouring an increased rate of wing growth relative to the rest of the body, thus making many tropical fledglings longer winged and better fliers than their temperate counterparts [2]. In mallard ducks (*Anas platyrhynchos*), selection for precocial leg use shifts growth to the hindlimbs early in development, prohibiting flight prior to reaching adult size [3]. In Trinidadian guppies (*Poecilia reticulata*), selection favouring increased investment in reproductive effort in high-predation (HP) environments results in pregnant females that are more distended at late-stage pregnancy, and thus suffer a relatively high performance detriment in maximum velocity and distance travelled, compared to pregnant low-predation (LP) females [4]. Also in the guppy, selection for improved feeding ability favours large guppy offspring in LP environments [5], and recent findings show that larger offspring are born more morphologically and functionally mature than their smaller counterparts [6,7]. Here, we use the Trinidadian guppy to investigate what specific changes in developmental rate or birth timing occur in order to produce the larger, more mature neonates.

Guppies are freshwater, live-bearing, lecithotrophic poeciliid fishes inhabiting the streams flowing from Trinidad's Northern Range Mountains. Waterfall barriers separate populations. Below waterfalls, guppies coexist with many predatory fishes, which exert strong selective pressures favouring the production of many, small offspring, a greater investment into reproduction and an earlier age of first reproduction [8,9]. Above waterfalls, guppies live mostly free from predation, but in high numbers, which leads to heavy competition over limited trophic resources [10,11]. LP life histories have evolved in favour of producing fewer, larger offspring, less total investment in reproduction and delayed age of first reproduction [12,13]. LP localities are always found upstream of HP localities and are separated from them by waterfalls that are barriers to the upstream dispersal of predators. Genetic data cluster the LP and HP sites within each river close to one another and well separated from the other rivers [14]. This clustering of HP and LP sites within each river and separation among rivers shows that adaptation to HP and LP environments occurred independently in each of them, or that they represent independent replicates in which guppies have adapted to these alternative environments.

In the Trinidadian guppy system, large offspring size repeatedly evolves in LP environments from HP (small offspring size) ancestors [8,9,15]. The mechanism by which larger offspring are produced in LP environments is through an increase in the amount of yolk provisioned by the mother to individual eggs, and an approximately 10% increase in gestation period [8,12,15–17]. An increase in yolk and longer gestation correlates with larger and more mature guppy offspring [6,7]. It has been found that LP offspring are born nearly fully ossified, while their HP counterparts are nearly entirely cartilaginous at birth [6,7]. Through a modest 15% increase in yolk provisioning [16], LP offspring are born 400% more ossified within the cranial skeleton [7]. The concomitant shift towards increased maturity (osteogenesis) with increased offspring size suggests that the LP descendants acquire later life-stage morphologies from their HP ancestors. But what specific changes in developmental rate or birth timing occur to produce such a phenotypic shift? Previous studies have limited quantification of maturation to neonatal age class only and focused on cranial (and limited axial) morphology [6,7]. Here, we measure maturation of the cranial, axial and appendicular musculoskeletal system from prenatal, neonatal and postnatal age classes (hereafter ‘perinatal window’) among five populations of Trinidadian guppy.

**Figure 1.**
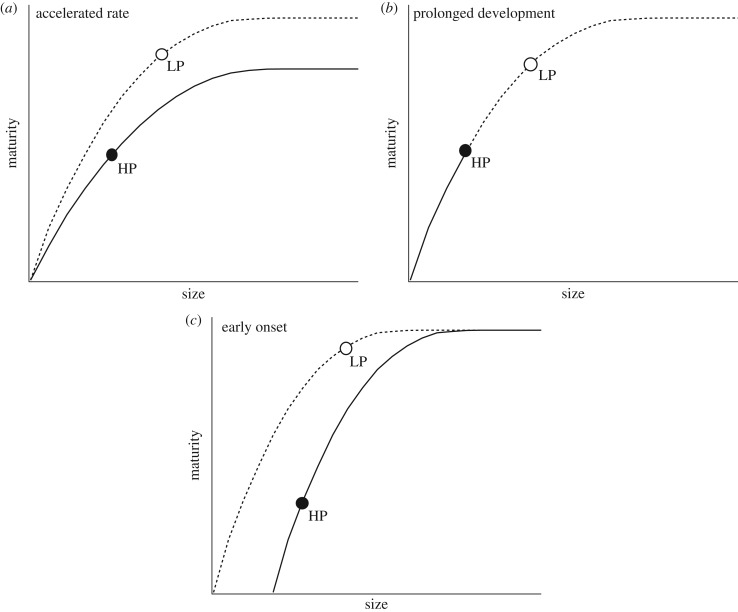
Developmental trajectories from embryo to late-stage juvenile. The evolution of offspring size at birth (neonatal size indicated by the solid and open dots) from small ancestor (HP; solid dot) to large descendant (LP; open dot) could be the product of three potential heterochronic scenarios. (*a*) Accelerated rate of development occurs when a steeper size–maturity relationship exists in the descendant. (*b*) Prolonged development (hypermorphosis) occurs when rates are similar, but the duration of development is prolonged in the descendant. (*c*) Early onset (pre-displacement) occurs when rates of development remain the same, but the process of development initiates earlier in the descendant. Here, maturation of the HP ancestor is assumed to begin later into prenatal growth.

There are three potential shifts in the rate or timing of development (i.e. heterochronies) that could produce the observed morphological evolution [18]. The first way would be by evolving accelerated maturation rates. If accelerated maturation rate is the dominant process, we predict the descendant LP populations will acquire bone and muscle maturity at a faster rate during the perinatal window compared to their ancestral HP counterparts (figure 1*a*). The hypothesis that prenatal developmental rates could be different is based on works showing that postnatal growth rates differ between the predation regimes, where HP offspring grow relatively fast and catch up to the size of LP juveniles by approximately 30 days post birth [19]. Alternatively, rates of somatic development could remain the same between descendant and ancestor, but growth and development of the descendant could extend over a longer period of time, as suggested by the longer gestation time of LP populations (figure 1*b*). If prolonged development is the dominant process, we would expect to observe a similar ontogenetic trajectory for all populations, but LP offspring will be born at later stages along this line (figure 1*b*). Finally, rates of growth and development might parallel between descendant and ancestor, but the onset of maturation in the descendant could shift to an earlier stage in gestation (figure 1*c*). In this final scenario, we would expect to observe a phase shift towards earlier bone and muscle development in descendant LP populations (figure 1*c*).

In order to test which of these three possible heterochronies best describes the evolution of the guppy offspring phenotype, we characterized the ontogenetic trajectory of growth and maturation among several populations that vary in offspring size at birth. We examined the maturation of the musculoskeletal system as a proxy for overall maturity, comparing five populations of Trinidadian guppies at the perinatal window. These populations were chosen to represent two independent origins of large offspring size. Additionally, the populations span a relatively wide gradient of predation [20] and offspring size, from large LP offspring to smaller HP to extremely small HP offspring. Although the cranial maturation of neonates has been described previously [7], the data are insufficient to reveal the underlying developmental processes yielding the observed shift in maturation among offspring. This dataset not only expands the window of investigation to include prenatal and postnatal forms (from five populations) but also includes the appendicular and post-cranial axial skeletons.

## 2. Material and methods

### (a) Collection and housing

This study compares five populations collected from two distinct drainages of Trinidad's Northern Range Mountains. Females from three populations were collected from the Caroni drainage, the major south slope confluence draining westward into the Atlantic: Aripo low-predation (Aripo LP), Aripo high-predation (Aripo HP) and Caroni high-predation (Caroni HP). Females from two additional populations were collected from the Yarra River on the north slope of the mountain range: Yarra low-predation (Yarra LP) and Yarra high-predation (Yarra HP). Previous studies have revealed that some populations are extreme in their HP phenotype (i.e. offspring are even smaller at birth than in other HP localities) [20], and this was the case between the Caroni HP population and the more intermediate phenotype of the Aripo HP population. An intermediate HP phenotype was not found in the Yarra drainage.

The predator communities differ between northern and southern slopes; the former contain mainly cichlid predators, while the latter contain primarily gobies [15]. The selective pressures have been shown to be similar between slopes: high predation selects for accelerated life histories, while high competition in LP sites selects for prolonged life histories (i.e. longer gestation times, longer interbrood intervals, later age of first reproduction, less overall investment in offspring) [8,9]. In guppies, as in most fishes, female size is not a significant predictor of offspring size [8,9,21,22], so female size was not controlled for, although all females were collected in a similar size range.

Pregnant female guppies were collected from the five focal populations and were returned to a field laboratory, where they were isolated into 2 l tanks. Upon parturition, each mother was promptly removed to mitigate potential cannibalism and loss of the brood. Broods were separated into five or fewer individuals per tank to standardize feeding amounts and other density effects [19,23]. Fish were fed twice daily, once on a diet of *Artemia* nauplii in the morning, and again on a diet of algae flakes in the evening. Tanks were housed within an open air laboratory facility and exposed to ambient light, which maintained 12 L : 12 D cycle.

Forty-two neonates, 21 prenatal and 31 postnatal guppies (94 total) were collected for morphological analysis. Neonates were collected at birth from at least four females per population. Postnatal juveniles were reared within the same 2 l tanks in which they were born and were fed twice daily. Prenatal individuals were obtained from gestating females. These near-term pregnant females were anesthetized by immersion into a buffered solution of 1 g l^−1^ tricaine methanesulfonate (Tricaine-S, Western Chemical Inc., Ferndale, WA, USA) and were then placed supine on a pillow made of cotton balls wrapped in Kim wipes. The female's abdomen was then incised from the cloaca cranially to the mid abdomen, using fine forceps and a sharp scalpel blade. The embryos were expelled with gentle pressure applied to the cranial aspect of the abdomen. These prenatal guppies and the mothers were then euthanized by overdose of tricaine. Specimens to be cleared and stained were fixed in 4% buffered paraformaldehyde (Sigma, St Louis, MO, USA) overnight and transferred to 70% EtOH for long-term storage. Specimens for immunohistochemistry were fixed in Dent's fixative: 80% Methanol (Fisher Scientific, Hampton, NH, USA; A412–500) and 20% dimethyl sulfoxide (Fisher Chemical, Hampton, NH, USA; D128–500) overnight and stained the following day. The first embryonic stage at which there is measurable uptake of alcian blue or alizarin red is around the flexion stage [24]; all prenatal embryos prior to this stage were not used in the analysis.

### (b) Staining

Bone and cartilage were differentially stained and the body was cleared to enable us to characterize and quantify differences in skeletal morphology associated with offspring size. Bone and cartilage stains were performed following the methods outlined in [7]. This staining allows for visualization of the internal skeleton and scale development (both total body coverage and scale growth rings). Specimens were visualized using a Nikon dissecting microscope (Nikon SMZ800 dissecting scope and Nikon DXM1200C digital camera).

To determine the degree to which cranial musculature is developing around the time of birth, neonates were immunostained with the muscle-specific antibody MF-20 (DSHB, Iowa, USA), visualized after performing HRP colour reaction (DAB chromogen kit, Biocare Medical, Concord, CA, USA) and the perimeter of the adductor mandibulae traced from a lateral view (for methods see [7]). Myomere development was also visualized using this colour reaction technique. Specimens were imaged in 1 : 1 glycerol : phosphate buffered saline solution under a dissecting microscope (Nikon SMZ800 dissecting scope and Nikon DXM1200C digital camera).

### (c) Analysis

Quantification of ossification within the skeleton was performed by identifying (and assigning a value to) the presence (1), partial presence (0.5) or absence (0) of alizarin red uptake within each of the following skeletal elements: angulo-articular, autopalatine, basihyal, branchiostegal rays, centra, ceratohyal, cleithrum, coracoid, dentary, epihyal, fin rays, frontal, hyomandibula, hypohyal, hypural, maxilla, operculum, pharyngeal jaws, prefrontal-lateral ethmoid, premaxilla, prevomer, quadrate, ribs, scapula, teeth and urohyal.

Ossification of cranial elements was presented as percentage of total skeleton ossified, which was quantified by determining the mean of the values for the presence (1), partial presence (0.5) or absence (0) of all 26 elements measured in the head skeleton. This type of averaging yields, for example, a skeleton with 13 elements fully ossified (13 × 1 = 13) and 13 elements partially ossified (13 × 0.5 = 6.5) an average of 75% ossification (19.5/26 × 100 = 75%).

Sequence of ossification was determined within the entire dataset by identifying the smallest standard length (SL) for which each element first appeared to uptake alizarin red. We also identify the SL above which each element is always ossified (based on more than 20 fish per element). At sizes between these two points, each element may be or may not be ossified.

The surface area of the superficial adductor mandibulae muscle group was measured using the ImageJ v.1.42 (National Institutes of Health, Bethesda, MD, USA). The stained muscle was outlined for each specimen to determine the area. These values were log-transformed and regressed against log SL to yield scaling relationships.

### (d) Statistical analysis

Values for adductor mandibulae area were log-transformed and regression lines (least squares) fitted against log SL to determine scaling relationships for each population. The resultant scaling coefficients were compared against expected isometric scaling values for each variable and against each population. One-way ANOVAs were performed on morphological variables with population as the factor. Where relevant, Tukey HSD post hoc tests were performed to identify means that are significantly different. Prior to performing an analysis of covariance, we tested for homogeneity by crossing standard length with population (SL*pop). If the interaction of SL*pop was not significant, we then ran the ANCOVAs with population as the factor and standard length (SL) as the covariate.

We predicted *a priori* that the rate of maturation of ossification would follow a sigmoidal growth trajectory. Sigmoidal growth curves were fitted to the data for each of the five populations. From these models, the maximum (± standard error) and growth rate (± standard error) values were compared to test for evidence of accelerated rate, prolonged development or early onset of morphological features. All statistical analyses were performed using the JMP v. 11 (SAS Institute, Cary, NC, USA).

## 3. Results

**Figure 2.**
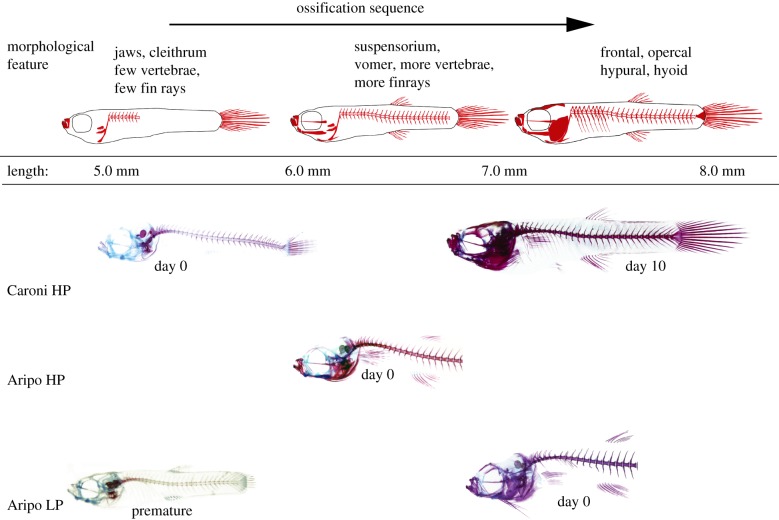
Illustration and representative images of guppies from three populations in the southern drainage aligned by SL. Day 0 individuals are neonates, collected on the day of birth. Postnatal Caroni day 10 individual and prenatal Aripo LP individual are also represented, and are aligned by size. The ossification sequence of the guppy proceeds along a standardized sequence, beginning with the jaws at 5 mm and terminating with the hyoid apparatus at 8.5 mm. The two Yarra populations show the same ossification sequence.

As expected from previous studies [6,9], neonatal size differed among populations, varying consistently with predation regime (figure 2). Mean LP standard lengths were 7.1 and 6.9 mm for the Yarra LP and Aripo LP drainages, respectively. Mean HP standard lengths were 6.2, 5.8 and 5.3 for Aripo HP, Yarra HP and Caroni HP, respectively. Offspring from LP localities were significantly larger than their HP counterparts (ANOVA: *F*_4,75_ = 56.01; *p* < 0.01). Despite these initial offspring size differences, all postnatal guppies converged on similar sizes by 30 days after birth (ANOVA: *F*_4,20_ = 1.38; *p* = 0.28).

**Figure 3.**
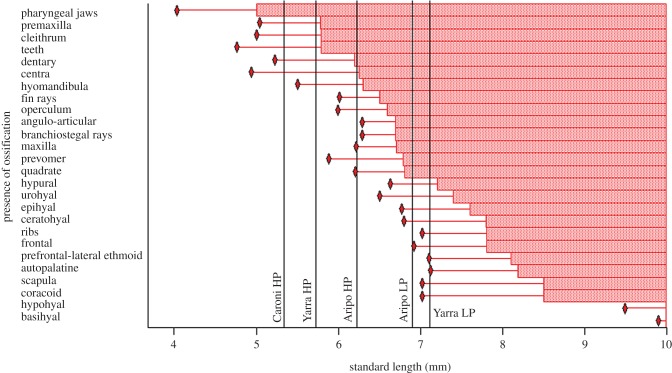
Ossification sequence of data compiled from five populations of Trinidadian guppy as a function of standard length (SL). Red diamonds indicate SL at which the first appearance of ossification was observed for each element. The narrow horizontal line indicates the size range over which each element may or may not be ossified. Solid bar indicates SL at which each element is always ossified. Black vertical lines indicate population mean SL at birth.

The first skeletal elements to display ossification were the pharyngeal jaws, which exhibited positive uptake of alizarin at sizes as small as 4 mm (figures 2 and 3). Elements involved in oral jaw feeding were next to ossify at 5 mm (figures 2 and 3). Elements involved in locomotion, such as parts of the vertebral centra and caudal fin rays, were next to ossify (SL 5.8 mm), followed by most of the cranial suspensorium (SL 6.0 mm), vomer (SL 6.5 mm), frontal bone (7.0 mm) and finally, the hyoid structures (SL 8.5 mm; figures 2 and 3). The specific order of ossification of the guppy skeleton was: pharyngeal jaws, premaxilla, cleithrum, teeth, dentary, centra, hyomandibula, fin rays, operculum, angulo-articular, branchiostegal rays, maxilla, prevomer, quadrate, hypural, urohyal, epihyal, ceratohyal, ribs, frontal, prefrontal-lateral ethmoid, autopalatine, scapula, coracoid, hypohyal, basihyal (figure 3).

Populations were significantly different in degree of ossification at birth (figure 2; ANOVA: *F*_4,89_ = 10.07; *p* < 0.0001). The smallest neonates, Caroni HP and Yarra HP, were born with 22 ± 0.1% and 21 ± 0.1% of their skeleton ossified, respectively, while the Aripo LP and Yarra LP neonates were born with 71 ± 0.1% and 66 ± 0.1% ossification, respectively (Aripo HP were born with 62 ± 0.1% ossification; Tukey HSD).

**Figure 4.**
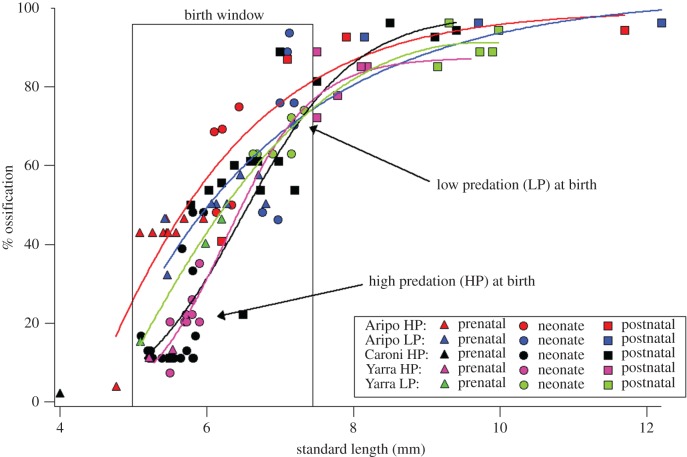
Per cent ossification of the skeleton among five populations within the perinatal window of development. Regression analysis fit sigmoidal models to each population (*R*^2^ values for each population: Aripo HP 0.76; Aripo LP 0.72; Caroni HP 0.69; Yarra HP 0.90; Yarra LP 0.84) and show onset and rate of ossification are no different among populations (see Results). At birth, populations display large differences in the degree of ossification along the trajectory. Neonates are represented by solid circles, postnatal juveniles by squares and prenatal embryos by triangles.

Across the perinatal window examined (approx. from 4 to 12 mm), guppies ranged from 0% to nearly 100% ossified, and offspring were born along the steepest part of this size–maturity relationship (figure 4). After size was accounted for, there was no significant difference in the degree of ossification among populations (ANCOVA: *F*_5,88_ = 2.37; *p* = 0.10). This indicates that the morphological differences observed among populations are owing to the differences in size at birth, and not owing to the population-level differences in developmental trajectories.

Degree of ossification varied with size among all five populations, but along a uniform ontogenetic trajectory throughout the perinatal window of maturation (figure 4). Populations developed along similar ontogenetic trajectories in terms of the onset and rate of ossification (figure 4). Sigmoidal regression analyses (*R*^2^ values for each population: Aripo HP 0.76; Aripo LP 0.72; Caroni HP 0.69; Yarra HP 0.90; Yarra LP 0.84) indicate that populations shared similar onsets (Aripo HP −6.96 ± 0.95; Aripo LP −5.84 ± 0.86; Caroni HP −7.83 ± 0.97; Yarra HP −6.32 ± 0.55; Yarra LP −7.77 ± 0.52) and rates of ossification (Aripo HP 1.70 ± 0.16; Aripo LP 1.53 ± 0.12; Caroni HP 1.68 ± 0.17; Yarra HP 1.38 ± 0.40; Yarra LP 1.74 ± 0.17).

**Figure 5.**
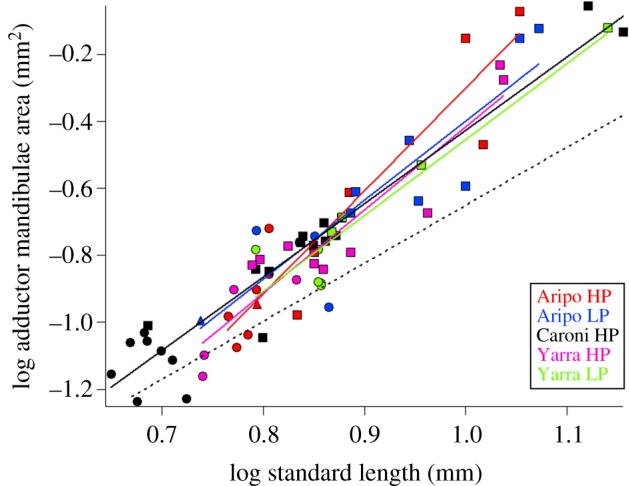
Scaling of adductor mandibulae muscle among five populations against size (SL). Log-transformed values indicate the scaling of all five populations exhibited similar positive allometry of the adductor mandibulae, such that larger offspring are born with relatively large jaw-closing muscles. Isometry was indicated by the dashed line. Neonates are represented by solid circles, postnatal juveniles by squares and prenatal embryos by triangles.

In concert with the maturing skeleton, scaling of the major jaw-closing muscle, the adductor mandibulae, reveals that larger offspring are more mature in muscular development. Within the observed perinatal window, area of adductor mandibulae scales with positive allometry among guppies, scaling as SL^2.5^ (figure 5). Positive allometric growth of the adductor mandibulae indicates that growth of this muscle outpaces growth of the body. If adductor mandibulae growth paralleled body growth, the data would fall along the dashed line in figure 5, which indicates isometry (SL^2.0^). Neonatal guppies were born along a positive allometric trajectory of muscle growth, such that larger offspring possess relatively large jaw-closing muscles. The difference in adductor mandibulae area was significantly different among neonates, where Caroni HP and Yarra HP neonates have significantly smaller muscle areas compared with their LP counterparts (ANOVA: *F*_4,25_ = 9.02; *p* < 0.01).

Similar to the maturation of the skeletal system, population-level differences in adductor mandibulae area can be accounted for by size at birth, rather than a change in developmental trajectory with predation regime. When size (SL) is not included in the analysis, populations vary significantly in adductor mandibulae area among neonates (ANOVA: *F*_4,25_ = 9.02; *p* < 0.01). After adjustment for differences in size, using SL as a covariate, no differences were observed among localities in adductor mandibulae area among neonates (ANCOVA: *F*_5,24_ = 0.74; *p* = 0.57). Size remains a significant predictor of adductor mandibulae area throughout the perinatal window (Regression analysis: *R*^2^ = 0.88; *F*_1,67_ = 471.17; *p* < 0.01). This ANCOVA and regression indicate that all five populations develop their jaw-closing muscles along a standardized growth trajectory, and larger LP neonates are born with relatively larger jaw-closing muscles than their smaller HP counterparts.

**Figure 6.**
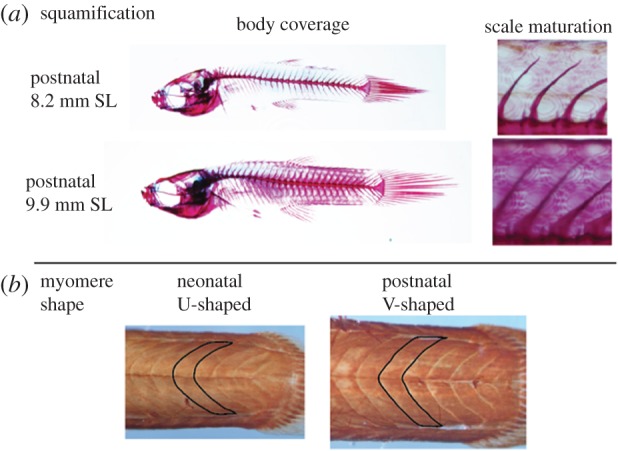
Maturation of some features continues into postnatal development. (*a*) Degree of squamification, in terms of body coverage and scale maturation, is more advanced in larger guppy juveniles. (*b*) Myomere shape shows that postnatal juveniles possess a more mature, V-shaped morphology, compared to their neonatal counterparts.

We observed additional morphological features that distinguished individuals into postnatal ontogeny. The degree to which scales matured in size and covered the body indicated that larger guppy juveniles possessed more mature levels of squamification (figure 6*a*). Additionally, myomere shape indicated that postnatal juveniles possess a more mature, V-shaped morphology, compared with their neonatal counterparts (figure 6*b*).

## 4. Discussion

We found that among five populations of the Trinidadian guppy, neonatal offspring span a size spectrum from small (5.3 mm SL) in some populations to large (7.1 mm SL) in other populations, but that developing guppies from all populations mature along a standard ontogenetic trajectory during the perinatal period. The degree and patterning of ossification and muscle growth suggests that these morphologies are acquired in a similar sequence and rate among developing guppies (figures 3–5), and that this trend is carried into postnatal ontogeny (figure 6). The order of ossification (figure 3) is similar to that reported from one population of Trinidadian guppy [24] and was invariant among the populations investigated, within our ability to distinguish among structures that appear at similar standard lengths. The major difference among populations is where, along this conserved sequence of maturation, the offspring are born (figures 2–5). The larger offspring have experienced more developmental events prior to birth and therefore appear to have undergone heterochrony, specifically hypermorphosis, or a prolonging of development (figure 1*b*) [25]. Similar to the disproportionate antlers of the Irish elk, this is a case of hypermorphosis, where rates of somatic development remain the same, but growth persists for a longer period of time [1,18,26]. Prolonging prenatal development along a standardized ontogenetic trajectory expands the window of birth that can be realized among guppy populations.

**Figure 7.**
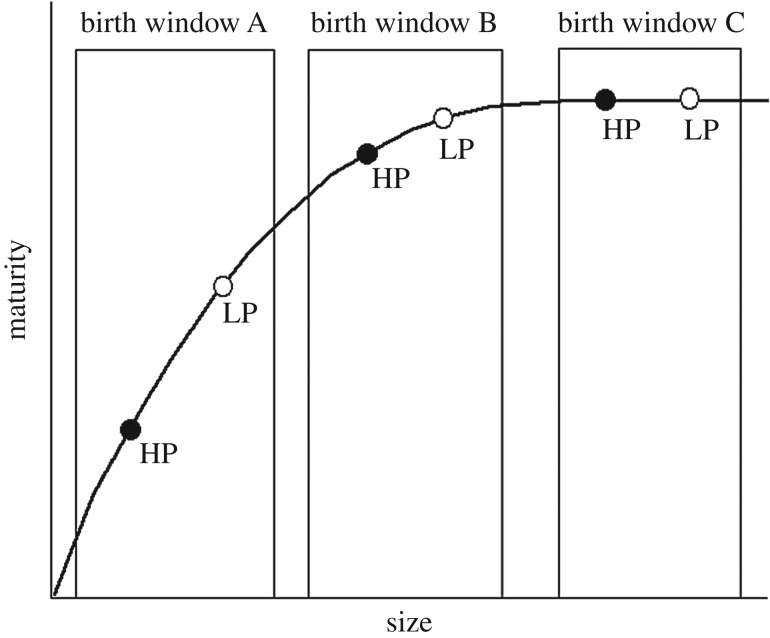
Hypothetical birth windows along a standardized trajectory of maturation reveal three distinct outcomes for an increase in offspring size. Many direct developing organisms are assumed to be born along the plateau of this relationship (window C), while guppy neonates are known to vary in the degree of maturity depending on size. In this study, we found that guppy offspring are born across birth window A, where a marginal increase in offspring size yields the largest increase in the degree of maturity. Birth window A might help facilitate the rapid evolution of the guppy phenotype in resource-limited environments.

We found that the birth window among populations falls along the steepest part of the guppy maturation curve (figure 7, birth window A), but other birth windows are hypothetically possible for this system. If we had found birth window C, then all guppy neonates would be born at the same developmental stage, just different sizes. Prior to this work, it was known that guppies are not born along the plateau of the size–maturity curve (birth window C), because both size and maturity of the feeding apparatus and tail region had previously been found to vary among neonates [6,7]. The degree to which size and maturity vary could place the birth window at either a shallow size–maturity relationship (figure 7, birth window B) or a steep relationship (figure 7, birth window A). Here, we find that among populations, guppies are born along the steepest part of the size–maturity curve (figure 7, birth window A). The musculoskeletal system of the entire body is undergoing the greatest rate of formation within this window, which means the offspring born at the top of the curve are substantially more mature with respect to the development of the musculoskeletal system than their smaller conspecifics born at the bottom (figures 4 and 5). The significance of this finding is that birth window A produces the greatest amount of morphological maturation for a given increase in offspring size.

### (a) Consequences for life-history evolution

The striking degree of morphological difference among neonatal guppies from HP versus LP populations offers clues to the causes, consequences and trade-offs associated with the evolution of guppy life histories. First, our results illuminate the balance of costs and benefits associated with the evolution of offspring size. By investing approximately 15% more yolk per egg [8], female guppies produce LP offspring that are 300–400% more mature (figure 4) and possess over 200% more jaw mobility at the intramandibular joint [7] compared to their HP conspecifics. The increase in mobility at the intramandibular joint has been shown to enhance feeding performance on encrusting substrates in poeciliids [27–29] and other benthic foraging fishes [30,31], and is implicated as a key morphological trait enhancing the competitive ability of LP offspring over their HP counterparts when resources are limited [7]. It is possible that, owing to the enhanced benefits of being born more mature, the marginal fitness gain for a given increase in per offspring investment by the female could be greater than what is expected from theory alone.

It is predicted that offspring fitness will increase linearly with per offspring investment [32–34], but in guppies the rate of increase in fitness appears larger. When competing over a limited amount of food, large LP guppy offspring have been shown to experience much greater fitness than small HP offspring [5]. An increase in foraging efficiency on encrusting substrates would benefit neonatal guppies born to environments with finite resources, and it is likely that the competitive ability of larger guppy offspring is enhanced by the greater degree of morphological maturity possessed among these neonates [7]. If maturity did not vary so tightly with size, it would perhaps be less adaptive to increase offspring size in environments of limited resources. Indeed, not only has selection favoured an average increase in the size of offspring born to LP environments over time, but it is also a consistent plastic response of female guppies when transported to low-resource environments [16].

The immediate phenotypic response of female guppies when deprived of food is an observed increase in interbrood interval (IBI), thereby producing larger offspring [16]. IBI is both the time to yolk up each ova and gestation time, thus it is not surprising that IBI is longer in the LP phenotype, where larger, yolkier eggs are produced [35]. LP fish have approximately 10% longer gestation periods [8,15], which simply produces larger and more mature offspring given that we do not find differences in prenatal growth or maturation rate among populations. The data presented herein provide a possible mechanism underlying this plastic response; the immediate adaptation from this shift in allocation of resources is observed in the function of the offspring produced, and not necessarily the prolonged IBI per se. Larger guppy offspring, born more mature, more competitive and experiencing higher fitness in resource-limited environments get over the first selective hurdle (surviving the first days of life) because of relatively high feeding performance. It is thus possible that the evolution of increased offspring size, which clearly is an adaptation to LP, high-competition environments [5], may represent a central, organizing feature of guppy life-history evolution because of the way it is integrated with other components of the life history.

There are many life-history traits that have been shown to evolve as guppies adapt to LP environments in addition to an increase in offspring size: reduced fecundity, longer IBI, decreased reproductive investment, delayed age of first reproduction and reduced growth rates [8,9,20]. Many of these traits, such as slower growth, are thought to be sufficient to explain adaptation to resource-limited environments [36], but the functional effects of producing more mature offspring at birth might be the first and most critical trait selected upon in highly competitive environments and could arguably increase selection for the rest of the LP phenotype. Because the suite of traits involved in adaptive life histories vary together, it is difficult to determine whether one trait drives the evolution of the life-history phenotype, or if each trait evolves independently, but at similar rates. What we can say here is that offspring size is a known critical trait and is the first trait selected upon in the life of newborn guppies (all other life-history traits, such as growth rate and delayed reproduction, follow afterwards). The finding herein that maturity varies widely across a standard birth window (figures 4–7), which correlates with enhanced fitness in LP environments [5], suggests that offspring size is perhaps the trait most relevant to near-term survival in a resource-depleted environment. The suite of guppy life-history traits that routinely evolve in LP environments may simply be the downstream product of selection acting on offspring size during the first, critical days of life.

## Data accessibility

Morphological data from prenatal, neonatal and postnatal guppies (from all five populations) have been deposited in the Dryad Digital Repository (http://dx.doi.org/10.5061/dryad.mn6pm) [37].
